# Prospective evaluation of a hydrogel spacer for rectal separation in dose-escalated intensity-modulated radiotherapy for clinically localized prostate cancer

**DOI:** 10.1186/1471-2407-13-27

**Published:** 2013-01-22

**Authors:** Franziska Eckert, Saladin Alloussi, Frank Paulsen, Michael Bamberg, Daniel Zips, Patrick Spillner, Cihan Gani, Ulrich Kramer, Daniela Thorwarth, David Schilling, Arndt-Christian Müller

**Affiliations:** 1 Department of Radiation Oncology, Eberhard Karls University Tübingen, Hoppe-Seyler-Str. 3, Tübingen, 72076, Germany; 2 Department of Urology, Eberhard Karls University Tübingen, Hoppe-Seyler-Str. 3, Tübingen, 72076, Germany; 3 Department for Diagnostic and Interventional Radiology, Eberhard Karls University Tübingen, Hoppe-Seyler-Str. 3, Tübingen, 72076, Germany; 4 Section for Biomedical Physics, Department of Radiation Oncology, Eberhard Karls University Tübingen, Hoppe-Seyler-Str. 3, Tübingen, 72076, Germany

**Keywords:** Prostate cancer, Intensity-modulated radiotherapy, Hydrogel spacer, Rectal toxicity, Dose-escalation

## Abstract

**Background:**

As dose-escalation in prostate cancer radiotherapy improves cure rates, a major concern is rectal toxicity. We prospectively assessed an innovative approach of hydrogel injection between prostate and rectum to reduce the radiation dose to the rectum and thus side effects in dose-escalated prostate radiotherapy.

**Methods:**

Acute toxicity and planning parameters were prospectively evaluated in patients with T1-2 N0 M0 prostate cancer receiving dose-escalated radiotherapy after injection of a hydrogel spacer. Before and after hydrogel injection, we performed MRI scans for anatomical assessment of rectal separation. Radiotherapy was planned and administered to 78 Gy in 39 fractions.

**Results:**

From eleven patients scheduled for spacer injection the procedure could be performed in ten. In one patient hydrodissection of the Denonvillier space was not possible. Radiation treatment planning showed low rectal doses despite dose-escalation to the target. In accordance with this, acute rectal toxicity was mild without grade 2 events and there was complete resolution within four to twelve weeks.

**Conclusions:**

This prospective study suggests that hydrogel injection is feasible and may prevent rectal toxicity in dose-escalated radiotherapy of prostate cancer. Further evaluation is necessary including the definition of patients who might benefit from this approach. Trial registration: German Clinical Trials Register DRKS00003273.

## Background

Radiation dose-escalation is a major issue in prostate cancer, since there is convincing evidence that cure rates indicated by biochemical disease-free survival and prostate cancer-specific survival depend on the radiation dose to the target [1]. The German national S3-guideline [2] as well as the European EAU guideline [3] recommend a dose of 74 Gy for patients with clinically localized prostate cancer regardless of risk groups, and state that higher doses are applicable and correlate with outcome. The proposed linear correlation of biochemical control and total radiation dose underlines the importance of dose-escalation for the prognosis [1]. However, increased radiation dose to the rectum results in dose limiting toxicity [4].

Advanced treatment delivery such as intensity-modulated radiotherapy (IMRT) and image-guided radiotherapy (IGRT) demonstrated a decrease in rectal toxicity compared to three-dimensional conformal radiotherapy (3D-CRT) with equal radiation doses [5,6]. However, dose-escalation, even performed with highly conformal dose delivery, led to increased side effects in all studies [4,7-9]. Doses to the anterior rectal wall increase with the prescribed dose to the prostate, independent of the techniques used for treatment planning and application. Further decrease of rectal doses with more advanced techniques appears unlikely, as the anterior rectal wall is frequently part of the high-dose planning target volume. As a consequence up to 20% of the patients develop acute and chronic rectal toxicity of grade 2 or higher after dose-escalated IMRT [5,6].

A recent technique for better sparing of the rectal wall is mechanical separation of the prostate and rectum by placement of a spacer. Several different approaches are currently under clinical investigation such as hyaluronic acid [10,11], collagen [12], biodegradable balloons [13] or polyethylene glycol (PEG) [14-16]. These approaches consistently led to lower rectal doses in planning studies. The application of a spacer in combination with high-dose-rate (HDR) brachytherapy for prostate cancer showed favorable acute toxicity [17]. Reduced side effects were also reported for rectal separation by transperineal injected collagen and prostate IMRT without radiation dose-escalation [12].

The current study reports the first prospective toxicity data of dose-escalated IMRT to 78 Gy in combination with rectal separation by a PEG-based medical device, and evaluates feasibility and acute toxicity.

## Methods

Eleven patients with histologically confirmed, organ confined (T1-2 N0 M0) adenocarcinoma of the prostate (Gleason score 6–7, PSA levels below 20 ng/ml) were enrolled in a prospective study for evaluation of acute and chronic toxicity of IMRT to 78 Gy to the target volume by using the hydrogel spacer SpaceOAR™ (SpaceOAR™ System, Augmenix Inc., Waltham, MA) for rectal separation. The choice for this PEG-based hydrogel compound was derived from the evaluation of biocompatibility, residence time and costs as discussed by Susil et al. [15]. The prospective study was approved by our institution’s ethics committee (Ethik-Kommission an der Medizinischen Fakultät der Eberhard-Karls-Universität, reference number 079/2011MPG23, study identification number in the German Clinical Trials Register: DRKS00003273). Written informed consent was obtained from all patients. Patients with a high risk of adhesions in the perirectal space, e.g. suffering from inflammatory bowel disease, chronic prostatitis and perianal disease or T3-tumors were not eligible.

All patients underwent prostate MRI (magnetic resonance imaging) to exclude extraprostatic spread. The injection of the hydrogel was performed in an outpatient setting using local anaesthesia and oral antibiotic prophylaxis. After transperineal needle insertion between the rectum and the Denonvillier fascia and hydrodissection with saline under ultrasound control, the hydrogel was injected. A subsequent MRI scan was performed to facilitate the radiation planning process by easy visualization of the hydrogel spacer. The distance created between prostate and rectum achieved by the spacer was measured at prostate apex, center and base. To avoid artifacts caused by different filling of seminal vesicles, the prostatic base was defined as prostate 3 mm below the origin of the seminal vesicles.

Radiotherapy was planned on the basis of three subsequent CTs (computed tomography) in the supine position with a slice thickness of 3 mm. The 3 CT datasets were registered with respect to the bony structures using the Treatment Planning Software (TPS) Oncentra Masterplan® (Theranostic GmbH, Solingen, Germany). Image fusion of the post-injection MRI and CT data sets for visualization of the spacer was performed using a mutual information algorithm. Clinical target volumes (CTV) and organs at risk (OAR) were contoured in each of the three CT data sets by two radiation oncologists (ACM, FP) with assistance of a specialized radiologist for prostate cancer (UK). The CTV included prostate only for low risk patients and an additional proximal 1-2 cm of seminal vesicles for intermediate risk patients. OARs comprised rectum extending from the anal verge to the rectosigmoid flexure, entire bladder, large and small bowel if present, bilateral femoral heads, penile bulb and skin.

From the 3 delineated contours for CTV, a single enclosing union was derived to account for interfraction organ motion and volume changes. Expansion of this union by 7 mm isotropically led to the coverage probability planning target volume (PTV_CP_). Similarly, OAR unions were created from 3 separately delineated contours. The prescribed dose for the PTV_CP_ was 5x2 Gy/week to a total dose of 78 Gy using a coverage probability approach based on an equivalent uniform dose (EUD) concept. The coverage probability approach consists of assigning individual coverage probabilities of the PTV_CP_ and the OARs to each voxel. The cumulative probabilities are then used as local weights in the cost function during IMRT optimization. As described previously, this treatment planning strategy provides robust IMRT plans and optimal rectal sparing in dose-escalated prostate IMRT [18]. Radiation doses to OARs were additionally evaluated by dose-volume-histogram (DVH) parameters.

IMRT treatment plans were generated with the software package Hyperion® (University of Tübingen, Tübingen, Germany) which uses a Monte Carlo dose engine. Serial constraints were implemented for bladder (k=8) and rectum (k=12) to reach a final maximum EUD of 60 Gy and 65 Gy, respectively [19]. Additional dose constraints for rectum were a V70 of 20% and a V75 of 15%, i.e. a percentaged rectal volume (V) receiving the dose of at least 70 or 75 Gy. IMRT treatment was delivered with a 15 MV linear accelerator (Elekta Synergy S, Elekta Oncology Systems®, Crawley, UK) equipped with a 4 mm multileaf collimator in a sliding window technique. The position of the prostate was regularly verified by conebeam CT according to an image-guidance protocol with an online intervention threshold of 3 mm to account for interfractional prostate motion and to monitor filling of rectum and bladder.

Planning CTs and radiotherapy were performed with a bladder-filling protocol and the use of laxatives. Patients with intermediate risk constellation were offered additional antihormonal therapy for 4–6 months. Acute toxicity was documented weekly during radiotherapy and three months thereafter according to RTOG (Radiation Therapy Oncology Group) classification [20]. The statistical analysis was performed with the software package SPSS 19 (SPSS Inc., Chicago, Illinois, USA). Distance between prostate and rectum was compared by the one-sided t-test for dependant variables.

## Results

The hydrogel spacer was successfully injected in ten of eleven patients treated at our institution from August 2011 to August 2012. Patient characteristics are summarized in Table 1. In the remaining patient, the Denonvillier space did not open during hydrodissection.

**Table 1:** Patients’ characteristics and clinical results

	**Age (years)**	**T-stage**	**Gleason-score**	**PSA pre-RT(ng/ml)**	**NCCN-risk-category**	**Max. GI-toxicity**	**Max. GU-toxicity**
Pat 1	67	1c	3+3	7.6	low	1	1
Pat 2	67	1c	3+4	9.9	intermediate	1	1
Pat 3	76	2b	3+4	4.4	intermediate	1	1
Pat 4	60	1c	3+4	5.8	intermediate	1	2
Pat 5	67	1c	3+4	5.1	intermediate	1	1
Pat 6	79	2a	4+3	9.9	intermediate	1	2
Pat 7	78	1c	3+4	13	intermediate	0	2
Pat 8	76	1c	4+3	9.2	intermediate	0	2
Pat 9	75	1c	4+3	3.9	intermediate	0	1
Pat 10	66	1c	3+3	8.5	low	1	2

Abbreviations

GI – gastrointestinal (RTOG).

GU – genitourinary (RTOG).

NCCN – national comprehensive cancer network.

Pat – Patient.

RT – radiotherapy.

**Figure 1:**
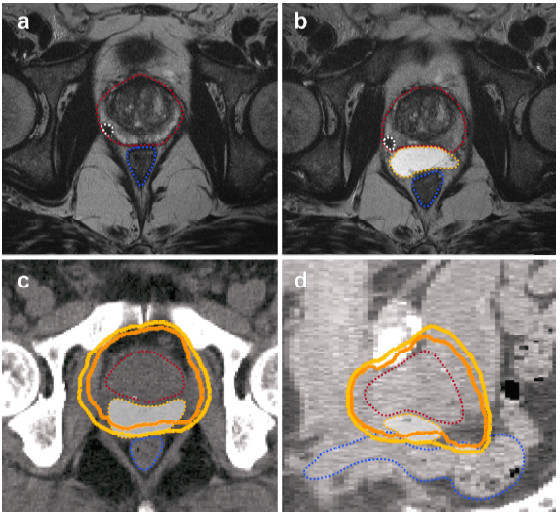
**Example of MRIs (T2 TSE) performed before (a) and after (b) injection of a hydrogel spacer.** The respective plan in axial **(c)** and sagittal view **(d)** with 70 Gy (light orange) and 74 Gy (orange) isodoses shows the rectal sparing with the use of the hydrogel spacer. Prostate (red), tumor (white), rectum (blue) and hydrogel spacer (yellow, white shading in CT scans) are indicated. *Abbreviation:* TSE=Turbo spin echo.

With the use of prophylactic antibiotics, no complications such as inflammation, urinary retention or other side effects occurred. Four of the eleven patients reported slight discomfort lasting for a few days post-injection. The hydrogel placement was correct in all injected patients, as shown in the subsequent MRI scans (example in Figure 1). The spacer reproducibly separated prostate and rectum throughout the whole interface (the difference of the rectoprostatic distance was significant, p<0.01; Table 2).

**Table 2:** Geometric results of Space OAR™ injection

	**Distance apex (mm)**			**Distance center (mm)**			**Distance base (mm)**			**Spacer volume (ml)**
	**w/o Spacer**	**with Spacer**	**Difference**	**w/o Spacer**	**with Spacer**	**Difference**	**w/o Spacer**	**with Spacer**	**Difference**	
Pat 1	0	13	**13**	0	18	**18**	0	18	**18**	14.3
Pat 2	0	7	**7**	0	14	**14**	4	13	**9**	5.1
Pat 3	0	11	**11**	0	14	**14**	0	10	**10**	12.4
Pat 4	1	14	**13**	0	11	**11**	2	16	**14**	12.1
Pat 5	1	7	**6**	1	14	**13**	2	18	**16**	10.5
Pat 6	1	10	**9**	3	17	**14**	2	13	**11**	17.1
Pat 7	1	10	**9**	1	8	**7**	3	13	**10**	16.4
Pat 8	2	11	**9**	1	15	**14**	1	16	**15**	9.7
Pat 9	0	15	**15**	1	22	**21**	0	18	**18**	9.1
Pat 10	0	2	**2**	0	15	**15**	1	18	**17**	10.7
**mean±SD**			**9±4**			**14±4**			**14±4**	11.7±3.6

Distance between prostate and rectum at three different anatomically defined points (base, center and apex of prostate) was evaluated before and after spacer insertion.

Abbreviations

Pat – patient.

SD – standard deviation.

w/o – without.

Dose-escalation was possible, prescribed doses and constraints for organs at risk were met in all patients. The mean EUD for the PTV_CP_ was 76.2 Gy corresponding to 78 Gy prescribed to the target volume. While high doses were administered to the spacer, the mean rectal dose was limited to 40.4 Gy (Table 3). Intermediate dose levels in the rectum represented by V40 reached a mean value of 55.0% (range 34.3%-73.2%). High dose levels were low as indicated by a mean rectal V75 of 2.0% (range 0.2-3.8%) and a V70 of 10.1% (range 1.7-16.0%).

**Table 3:** Radiation dose parameters

	**PTV**_**CP**_	**Spacer union**	**Rectum union**				
	**EUD (Gy)**	**Mean dose (Gy)**	**Mean dose (Gy)**	**V75 (%)**	**V70 (%)**	**V65 (%)**	**V40 (%)**
Pat 1	75.8	72.8	38.0	0.2	1.7	7.9	43.4
Pat 2	76.8	75.9	42.0	3.8	16.0	24.7	58.5
Pat 3	76.3	74.2	41.4	2.8	11.3	21.3	56.8
Pat 4	75.7	72.3	40.4	0.7	5.0	14.4	57.1
Pat 5	76.6	74.1	42.0	3.2	11.2	19.0	58.5
Pat 6	75.4	72.9	40.5	3.7	14.9	23.8	50.9
Pat 7	75.8	73.2	47.8	1.1	13.1	24.4	73.2
Pat 8	76.9	77.8	29.5	1.1	7.2	11.7	34.3
Pat 9	76.6	75.6	41.3	3.1	11.8	19.8	60.6
Pat 10	75.7	71.9	40.6	0.5	8.8	18.7	56.4
**mean±SD**	**76.2±0.51**	**74.1±1.8**	**40.4±4.3**	**2.0±1.4**	**10.1±4.2**	**18.5±5.3**	**55.0±9.9**

Dose coverage in the PTV was evaluated based on the EUD in the PTV_CP_ (union of three single CTVs with a 7 mm margin weighted by the coverage probability). Rectal doses were described by DVH-parameters.

Abbreviations

DVH – dose volume histogram.

EUD – equivalent uniform dose.

Gy – Gray.

Pat – patient.

PTV_CP_ – coverage probability-planning target volume.

SD – standard deviation.

V – volume receiving respective radiation dose in Gy or more.

**Figure 2:**
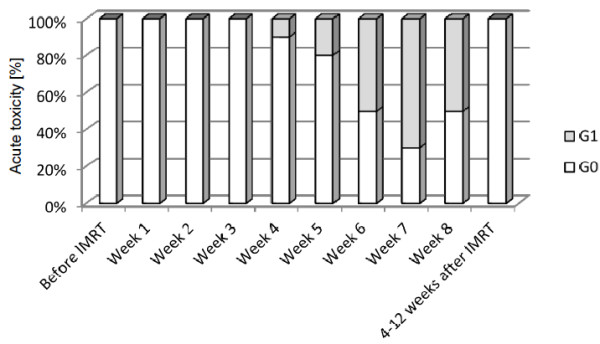
**Acute rectal toxicity.** Acute rectal toxicity was measured at baseline, weekly during IMRT and 4–12 weeks after treatment according to the RTOG-criteria. * Two patients were graded as RTOG G1 only due to mucous discharge, but not due to any other rectal symptoms. *Abbreviation:* RTOG=Radiation Therapy Oncology Group.

Acute rectal toxicity was mild, as shown in Figure 2. Five patients were classified as having RTOG grade 1 rectal toxicity in the last week of radiotherapy. Stool frequency had changed in two patients, no patient experienced new urge-symptoms or fecal incontinence. Side effects resolved completely within four to twelve weeks. Genitourinary side effects occurred with grade 1 in five patients and grade 2 in five patients.

## Discussion

This is the first report on prospective toxicity data for dose-escalated IMRT to 78 Gy with the use of a spacer for prostate-rectum separation in clinically localized prostate cancer.

The insertion of any spacer in the Denonvillier space creates a distance between prostate and rectum that allows calculation of dose-escalated radiation treatment plans, without exceeding accepted dose restrictions to the rectum. This has also been demonstrated by other groups [12,14,15]. Regarding constraints of current dose-escalated prostate cancer trials such as RTOG 0815, the achieved maximal rectal V70 of 16.0% and V75 of 3.8% were clearly below accepted rectal constraints such as V70 of 25% and V75 of 15%, derived from a recent analysis of six studies [21]. Our study was able to demonstrate the applicability of dose-escalated IMRT with limited radiation doses to the rectum. The high dose in the spacer volume shows the significance of the created distance for the rectal dose reduction. The mean rectal V70 of 10.1% was in line with previously published data of 4.5% in the cadaver planning study [15] and 7.5% in the first clinical planning study using the SpaceOAR™ system [22].

No significant side effects occurred in the first ten patients undergoing hydrogel injection on an outpatient basis. Four patients reported slight discomfort directly after the injection. As discussed by Vordermark et al. [23], side effects of the injection will be followed prospectively. The hydrogel injection was not feasible in one patient. In this case, the Denonvillier space did not open during hydrodissection, presumably due to adhesions. However, the patient did not have a history of inflammation in the perirectal or prostatic region. This indicates that this procedure cannot be performed in all patients.

For comparison of different IMRT fractionation schedules with regard to rectal toxicity, we calculated equivalent doses with 2 Gy per fraction with an α/β of 4.8 Gy, which was described for late rectal toxicity based on RTOG 94–06 [24]. Toxicity results are available for three IMRT dose schedules, as summarized in a recent review [25]. Zelefsky et al. reported late rectal toxicity grade 2 or higher (CTC-criteria) of 1.6% after 8 years. Patients had been irradiated with 81 Gy in 1.8 Gy fractions (equivalent dose 78.6 Gy) [26]. A hypofractionated regimen (2.5 Gy single-dose to 70 Gy, equivalent dose 75.1 Gy) led to acute rectal toxicity grade 2 or higher (RTOG criteria) in 7%, and to late toxicity grade 2 or higher in 6% (RTOG criteria) of the 400 patients treated from 2001–2005, with particular attention being paid to limit the rectal V70 [27]. Patients treated to a median dose of 75.6 Gy in 1.8-2.0 Gy fractions were reported to have acute rectal toxicity of grade 2 or higher (RTOG criteria) in 50%, leading to 24% with late rectal toxicity of grade 2 or higher (RTOG criteria) [28]. In contrast to this data, Noyes et al. reported no acute and late rectal toxicity (RTOG and EORTC criteria) with IMRT to 75.6 Gy in 1.8 Gy per fraction (equivalent dose 73.4 Gy) after transperineal collagen injection [12]. All results point towards a significant decrease in acute rectal toxicity by rectal separation. In line with these results, no grade 2 acute rectal toxicities occurred in our study with dose-escalated IMRT to 78 Gy (2 Gy/fraction). Regarding late fecal incontinence, which has a major impact on quality of life [29], a recent analysis showed a strong correlation with V40 of the rectum and acute toxicity. After 3D-CRT, 3.1% of 550 patients experienced new fecal incontinence. The authors found V40≥80% to be the best predictive parameter [30]. None of our patients met this criterion with the use of the hydrogel spacer, and fecal continence was not altered during radiotherapy. Thus, reduced frequency and severity of late fecal incontinence might be achievable with the use of SpaceOAR™.

## Conclusions

Our prospective data firstly show very low toxicity of dose-escalated IMRT with rectal separation by the use of a hydrogel spacer. The decrease in rectal dose was associated with only mild rectal acute toxicity (no grade 2 or higher) which completely resolved after three months. This may result in a low rate of late toxicity. Overall, this prospective study suggests that hydrogel injection is feasible, leads to low rectal acute toxicity and may therefore prevent rectal late effects in dose-escalated radiotherapy of prostate cancer. Further evaluation is necessary to define which patients might benefit from this approach.

## Competing interests

On behalf of all authors, the corresponding author states the following: Augmenix Inc. provided ten hydrogel spacers for the patients.

## Authors’ contributions

FE: Substantial contributions to interpretation of data, drafting and revising the article and final approval. SA: Substantial contributions to data acquisition, revising the article critically and final approval. FP: Substantial contributions to conception, revising the article critically and final approval. MB: Substantial contributions to conception, revising the article critically and final approval. DZ: Substantial contributions to conception, analysis and interpretation of data, drafting the article and final approval. PS: Substantial contributions to interpretation, revising the article critically and final approval. CG: Substantial contributions to interpretation, revising the article critically and final approval. UK: Substantial contributions to data acquisition, revising the article critically and final approval. DT: Substantial contributions to data acquisition, interpretation of data and final approval. DS: Substantial contributions to data acquisition, revising the article critically and final approval. ACM: Substantial contributions to acquisition, analysis and interpretation of data, drafting the article and final approval. All authors read and approved the final manuscript.

## Pre-publication history

The pre-publication history for this paper can be accessed here:

http://www.biomedcentral.com/1471-2407/13/27/prepub
